# Large-Scale Genomic Analysis of SARS-CoV-2 Omicron BA.5 Emergence, United States

**DOI:** 10.3201/eid3113.240981

**Published:** 2025-05

**Authors:** Kien Pham, Chrispin Chaguza, Rafael Lopes, Ted Cohen, Emma Taylor-Salmon, Melanie Wilkinson, Volha Katebi, Nathan D. Grubaugh, Verity Hill

**Affiliations:** Yale School of Public Health, New Haven, Connecticut, USA (K. Pham, C. Chaguza, R. Lopes, T. Cohen, E. Taylor-Salmon, N.D. Grubaugh, V. Hill); Yale School of Medicine, New Haven (E. Taylor-Salmon); Centers for Disease Control and Prevention, Atlanta, Georgia, USA (M. Wilkinson, V. Katebi); Yale University, New Haven (N.D. Grubaugh)

**Keywords:** COVID-19, Omicron BA.5, respiratory infections, severe acute respiratory syndrome coronavirus 2, SARS-CoV-2, SARS, coronavirus disease, coronavirus, phylogenetics, disease control, viruses, zoonoses, United States

## Abstract

The COVID-19 pandemic has been marked by continuous emergence of novel SARS-CoV-2 variants. Questions remain about the mechanisms with which those variants establish themselves in new geographic areas. We performed a discrete phylogeographic analysis on 18,529 sequences of the SARS-CoV-2 Omicron BA.5 sublineage sampled during February–June 2022 to elucidate emergence of that sublineage in different regions of the United States. The earliest BA.5 sublineage introductions came from Africa, the putative variant origin, but most were from Europe, matching a high volume of air travelers. In addition, we discovered extensive domestic transmission between different US regions, driven by population size and cross-country transmission between key hotspots. We found most BA.5 virus transmission within the United States occurred between 3 regions in the southwestern, southeastern, and northeastern parts of the country. Our results form a framework for analyzing emergence of novel SARS-CoV-2 variants and other pathogens in the United States.

SARS-CoV-2, the causative virus of the COVID-19 pandemic, has demonstrated the ability to evolve into novel variants. The Omicron (B.1.1.529) variant, detected in late 2021 in southern Africa, was deemed a variant of concern by the World Health Organization and soon became dominant in the United States and worldwide (*1*). Omicron lineages are defined by ≈60 mutations, 32 of those in the spike protein, that have granted evolutionary advantage over co-circulating variants because they enhance intrinsic transmissibility and immune escape (*1*–*4*). New Omicron sublineages, as well as recombinants, have subsequently emerged (*5*,*6*). Furthermore, the complex mosaic of immunity in the human population, likely caused by different levels of vaccination or previous infection, indicates the landscape for SARS-CoV-2 variant emergence has changed since the start of the pandemic. With ongoing variant emergence, changing patterns of spread must be elucidated, because those patterns have considerable implications in prevention and mitigation plans.

Recent advances in virus sequencing and phylogenetics has enabled the timely use of large-scale phylogenetic analyses to determine SARS-CoV-2 dynamics (*7*). Studies have been conducted globally, including in Brazil (*8*), The Gambia (*9*), and New Zealand (*10*), to explore the origins, emergence, and dynamics of SARS-CoV-2 variants. In the United Kingdom, multiple analyses of national-level spread from major population centers have been conducted, showing early spread from the origin(s) of introduction and the seeding and subsequent local transmission to new locations (*11*–*14*). Furthermore, studies in the United States have shown the increased risk for virus importation among states compared with international origin (*15*), the importance of superspreading events promoting early transmission (*16*), and effects of international introductions of the Alpha variant (*17*).

We used a Bayesian discrete phylogeographic framework to determine the introduction and spread of a novel SARS-CoV-2 lineage into different regions of the United States. We focused on Omicron sublineage BA.5 during its global emergence period within the first 6 months of 2022 because of its rapid national spread, long-term persistence, and public health importance (Figures 1, 2). Omicron sublineage BA.5 established itself during times of lower SARS-CoV-2 incidence and remained prominent until the end of 2022 (Figure 1) (*18*). However, BA.5 never achieved complete dominance in the United States, co-circulating instead with other major Omicron sublineages, such as BA.2.12.1, BA.4, and XBB.1 (*5*). Moreover, BA.5 dissemination occurred on the background of a highly immune population because of vaccination and previous infections with other Omicron sublineages (*5*,*19*). Newer variants are likely to be introduced onto a similar immune landscape; thus, the dynamics of BA.5 introductions and dissemination offer a useful case study for how new lineages might spread across the United States. Furthermore, because most social and travel restrictions have been lifted and data streams have become more limited, clarifying within-country spread will enable targeted surveillance activities in the future. 

**Figure 1:**
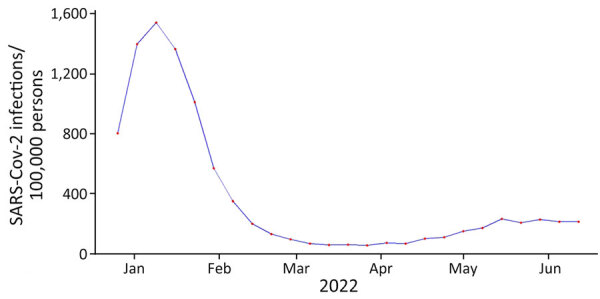
Number of estimated weekly SARS-CoV-2 infections in study of large-scale genomic analysis of Omicron BA.5 emergence, United States, January 2022–June 2022. Source: https://covidestim.org

**Figure 2:**
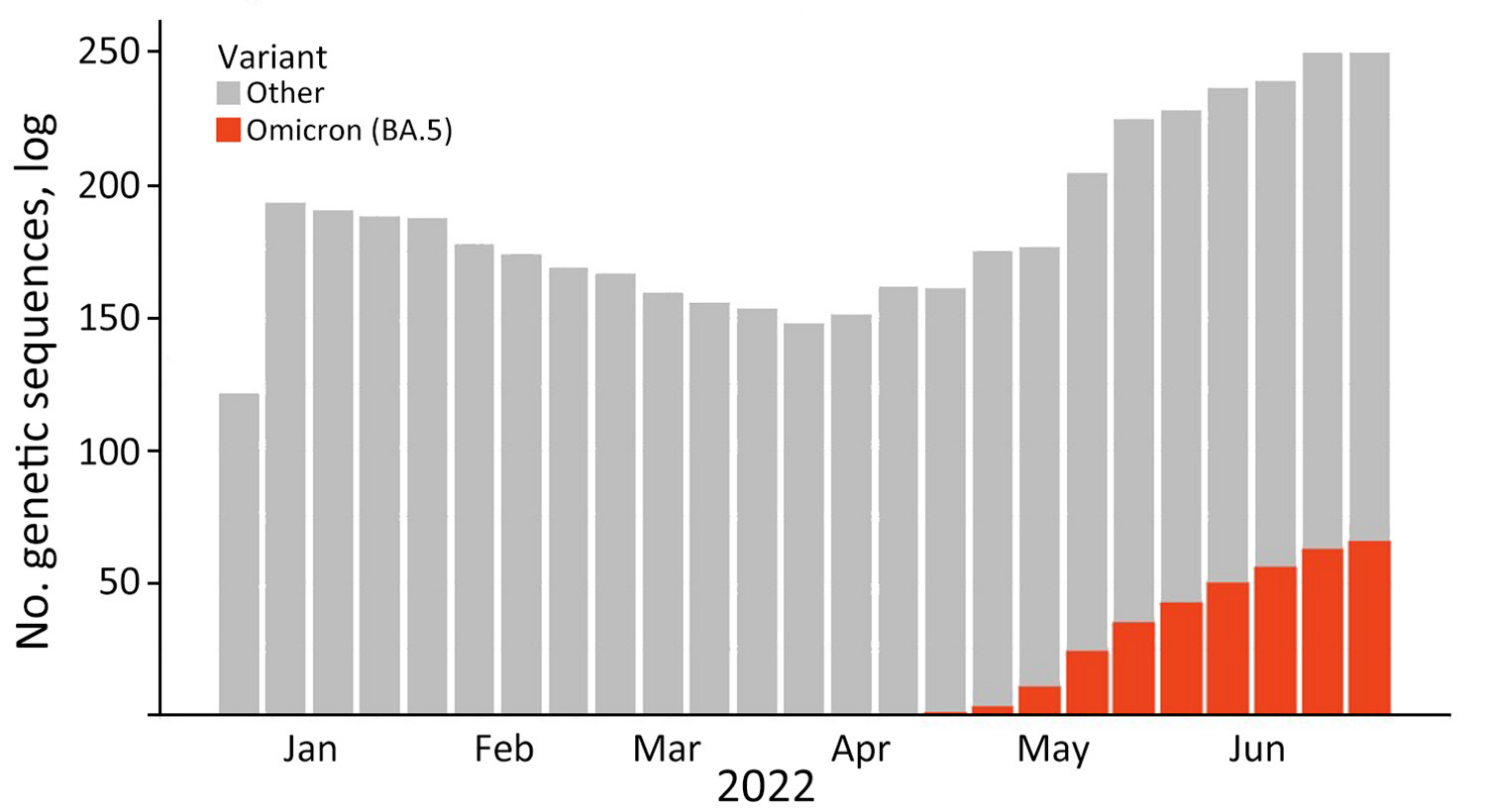
SARS-CoV-2 variant frequency during January–June 2022 in study of large-scale genomic analysis of Omicron BA.5 emergence, United States.

## Methods

### Dataset Generation

To define our study period, we balanced having a large enough time period to cover key events with avoiding an intractably large final dataset. Therefore, we compared the frequencies of Omicron BA.5 with other variants in each continent and selected the week for which every continent had a BA.5 frequency of >25% (week commencing June 13, 2022). That cutoff is somewhat arbitrary, but the speed of BA.5 spread on a continental level meant that changing the threshold only resulted in a few weeks’ difference either way (e.g., changing it to 50% added 2 weeks to the dataset; changing to 10% resulted in 1 week less).

We assembled a dataset of BA.5 whole-genome sequences sampled in the United States and globally during the inferred emergence period, estimated to be during February–June 2022. First, we downloaded all sequences that had complete location and collection date metadata from GISAID (https://www.gisaid.org) and had the BA.5 pango lineage designation (*20*). We then used Nextclade (*21*) to filter for low-quality control score and genome coverage of <70%. To mitigate sampling bias, we categorized global BA.5 data by continent. 

**Figure 3:**
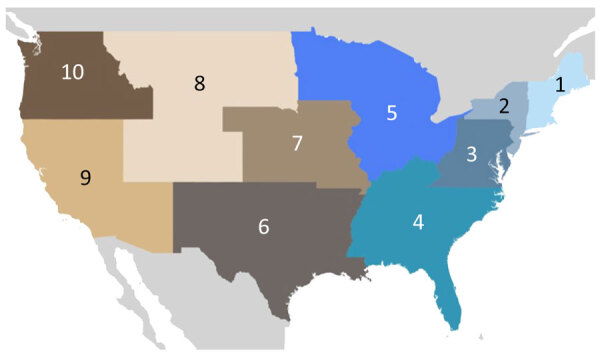
Ten regions of the United States evaluated in large-scale genomic analysis of SARS-CoV-2 Omicron BA.5 emergence. Regions have been designated by the US Department of Health and Human Services (https://www.hhs.gov/about/agencies/regional-offices/index.html).

Within the United States, the genomic surveillance policy is largely decided by the individual state, causing potential bias in data from each region (*22*,*23*). To ameliorate that disparity, we divided the country into 10 regions according to the locations of the 10 regional offices of the US Department of Health and Human Services (DHHS) (Figure 3). To account for possible selection bias from that heterogeneity, we subsampled the full dataset in 1-week windows proportional to the population of each region. We chose to use population because case counts are also biased between and within countries (especially those as large as the United States) because of varying availability of resources and case definitions. We felt this choice was appropriate for SARS-CoV-2 because so much of each country’s population was infected; thus, in this specific case, we decided that population was a less biased metric on which to base our subsampling scheme than case counts. Specifically, we used the population proportion of the region, either global continent or US region, and multiplied by the total number of BA.5 genomes to find 1 fixed number of genomes (selected every week for that region). The final dataset selected for analysis consisted of 18,529 sequences, 9,350 from the United States and 10,258 from non-US countries (Table). For the emergence period, the earliest sample was collected on February 25, 2022, whereas the latest sample was collected on June 19, 2022.

**Table:** Final genomic sequence dataset for BA.5 discrete phylogeographical analysis in study of SARS-CoV-2 Omicron BA.5 emergence, United States

Locations	No. sequences
Global	
Africa	1,579
Asia	3,273
Europe	1,589
North America	1,281
Oceania	455
South America	1,388
US regions*	
1	389
2	1,050
3	637
4	1,814
5	1,339
6	1,029
7	314
8	376
9	1,637
10	379

*Regions are shown in Figure 3.

### Phylogeographic Analysis

We performed multiple sequence alignments by using the Nextclade tool, Nextalign (*21*), and Wuhan-Hu-1/2019 as the reference genome (GenBank accession no. MN908947.3). We then constructed a maximum-likelihood phylogenetic tree by using IQ-TREE version 2.2.2 (*24*), the Hasegawa-Kishino-Yano nucleotide substitution model (*25*), and outgroup rooting on the MN908947 reference genome. We assessed the temporal signal by using TempEST version 1.5.3 (*26*) and found the timeframe for the dataset was too short to have a strong temporal signal (Appendix Figure 1). We were still able to prune molecular clock outliers (*27*) by using jclusterfunk version 0.0.25 (https://github.com/snake-flu/jclusterfunk).

Because of the large size of the genomic dataset, we used the alternative tree likelihood function in BEAST version 1.10.4, which was developed for efficient estimation of large phylogenies (*13*,*28*). We used maximum-likelihood trees described previously for the topologic estimation and time-calibrated those trees approximately by using TreeTime version 0.9.4 (*29*) to reduce the percentage of states that needed to be discarded for burn-in.

Because of the low temporal signal in the dataset, we fixed the clock rate at 8 × 10^–4^ substitutions/site/year, as previously described (*30*–*32*). We used the nonparametric Skygrid coalescent model (*33*) with 23 grid points defined according to approximately equal intervals within the global emergence period. We ran 2 Markov chain Monte Carlo chains for 1 billion iterations each to ensure convergence with the same part of the posterior distribution. We used Tracer version 1.7.1 d to assess convergence after run completion and discarded 10% of Markov model states for burn-in (*34*).

We chose 1 random tree from the post–burn-in posterior distribution from the previous analysis to use as the fixed tree in a discrete trait analysis. We analyzed 2 separate geographic scales: 1 analysis at the global level, which included 6 continents (Africa, Asia, Europe, North America without the United States, Oceania, and South America) and the United States as a country; and 1 analysis at the US national level, which included 10 DHHS regions using the continental dataset as background global context. In both analyses, we used an asymmetric continuous-time Markov chain to estimate transition rates between locations. Each chain ran for 2 million states; we discarded 10% of Markov model states for burn-in.

For the international analysis, we used custom Python scripts to estimate the average number of introductions across each tree in the posterior distribution and then selected a final tree closest to that average number. For the domestic analysis, we chose a random tree in the posterior distribution to maintain stability of clades within the United States across the analysis. We defined an introduction event as the point in which a node is in a different location than its parent, either originating from another continent into the United States (for international introduction) or between US regions (for domestic analysis). We did not account for reintroduction within the same clade; thus, once the location changed to the United States, that clade was counted as only 1 introduction event. If a node in 1 subtree coincided with a node in another subtree, we only counted the node that had the older root and eliminated the other. We determined the size of an introduction to be the number of sequences that immediately followed a change in location within a node. We estimated the time of introduction as halfway between the first US/domestic location node and its parent. We generated figures by using custom Python scripts and trees by using the Baltic Python package (https://github.com/evogytis/baltic).

To examine drivers of BA.5 domestic spread, we constructed a linear regression model that incorporated geographic proximity and population. Within the model, the proportion of directional domestic introductions between a pair of US regions was the outcome; the 2 independent variables were the binary neighboring relationship between that pair and the numeric total population of the 2 regions. We obtained population data from the US Census Bureau (https://www.census.gov).

### Travel Data

To examine possible factors affecting BA.5 spread in different US regions, we collected data for monthly international and domestic air travel into US states during February–June 2022 (*34*). Those data were adjusted air passenger estimates, sampled according to ticket sales and reporting from airline carriers and assumed to represent 100% of the market. Adjusted travel volume represents the aggregate number of passenger journeys, not necessarily unique persons. We defined passenger journeys as airline transport between original embarkment and disembarkment in the United States. Both direct and indirect (i.e., connecting) flights were included.

### Data Availability

The flight travel volume data were provided by OAG Aviation Worldwide Ltd. OAG Traffic Analyser, version 2.6.1 (http://analytics.oag.com/analyser-client/home; accessed 2023 Apr 24). The data were used under the US Centers for Disease Control and Prevention license for the current study and so are not publicly available. The authors are available to share the air passenger data upon reasonable request and with the permission of OAG Aviation Worldwide Ltd.

We obtained all genomic data from GISAID (acknowledgements table at https://doi.org/10.55876/gis8.240620dg). The XML files and outputs from the BEAST analyses are also available (https://github.com/grubaughlab/2025_paper_BA.5_United-States).

## Results

### International Introductions of Omicron BA.5 Sublineage into the United States

**Figure 4:**
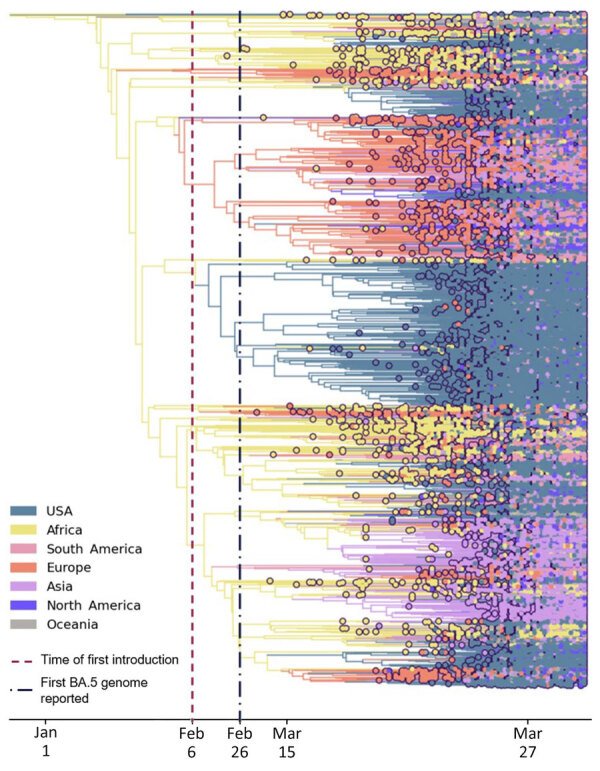
Time-scaled phylogeographic analysis of SARS-CoV-2 Omicron BA.5 sequences in the United States during January–June 2022. Analysis of BA.5 emergence was conducted by using 18,529 sequences collected globally and in the United States. Purple dotted line indicates the inferred date of the first introduction. The blue dotted line indicates the first sample of BA.5 sequenced in the United States. Colors indicate origin of the BA.5 variant.

**Figure 5:**
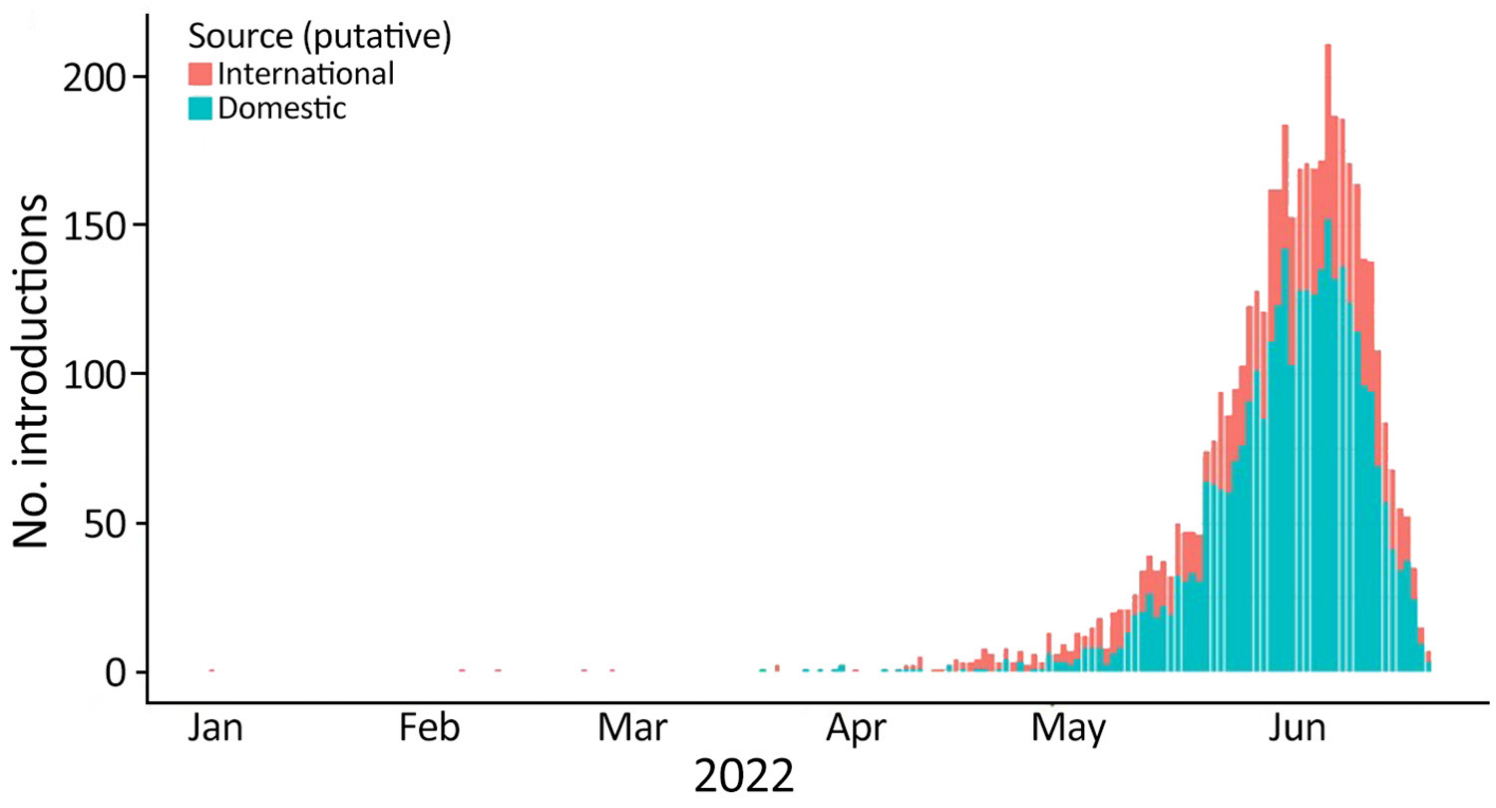
Numbers and timeline of domestic and international introductions of SARS-CoV-2 Omicron BA.5 in the United States during January–June 2022.

**Figure 6:**
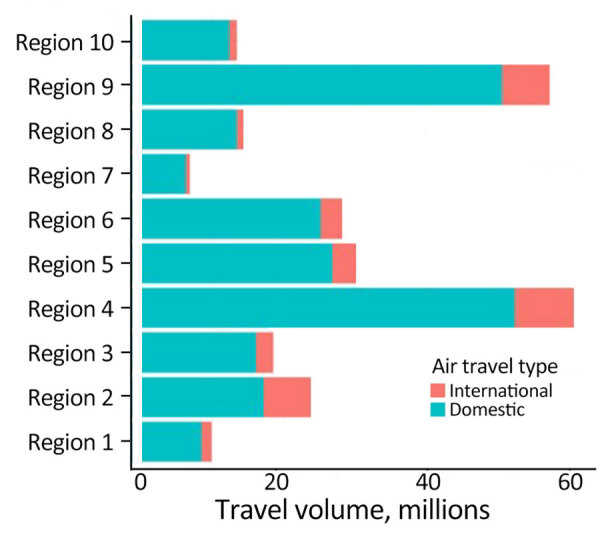
Air travel volume into different regions of the United States in study of large-scale genomic analysis of SARS-CoV-2 Omicron BA.5 emergence, January–June 2022. Domestic and international air travel volume are indicated. Regions designated by the US Department of Health and Human Services are shown in Figure 3.

**Figure 7:**
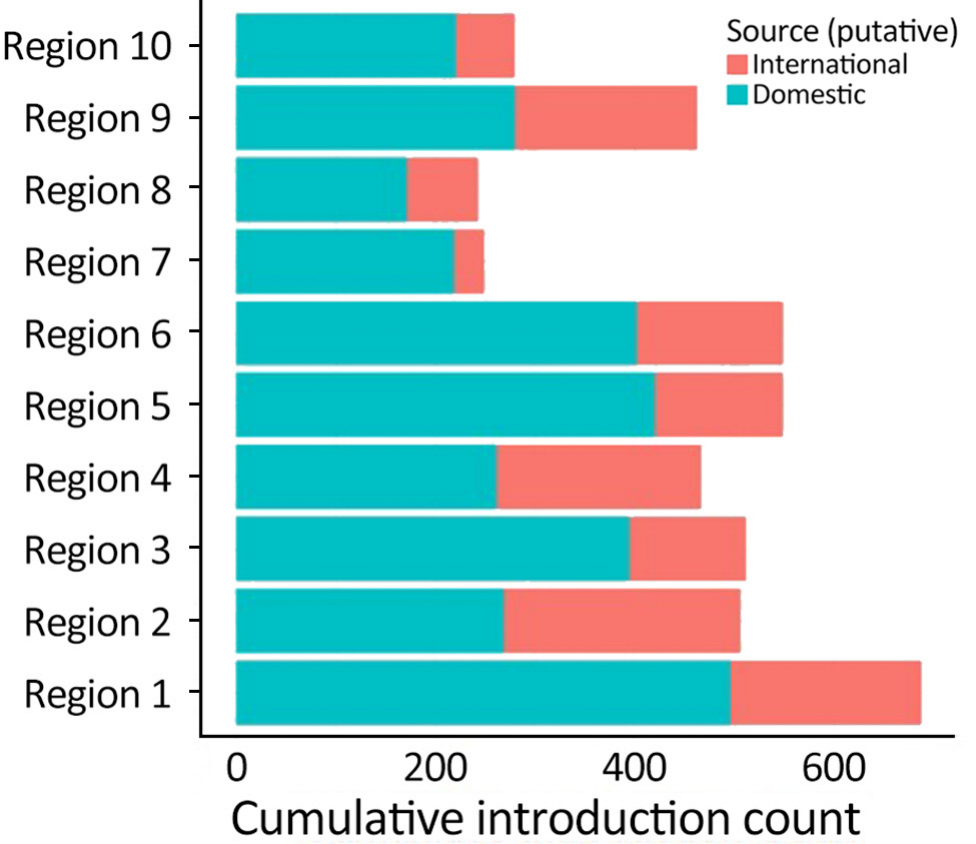
Total number of introductions of Omicron BA.5 into regions of the United States in study of large-scale genomic analysis of SARS-CoV-2 BA.5 emergence, January–June 2022. Cumulative numbers during the study period are indicated according to domestic or international origin. Regions designated by the US Department of Health and Human Services are shown in Figure 3.

**Figure 8:**
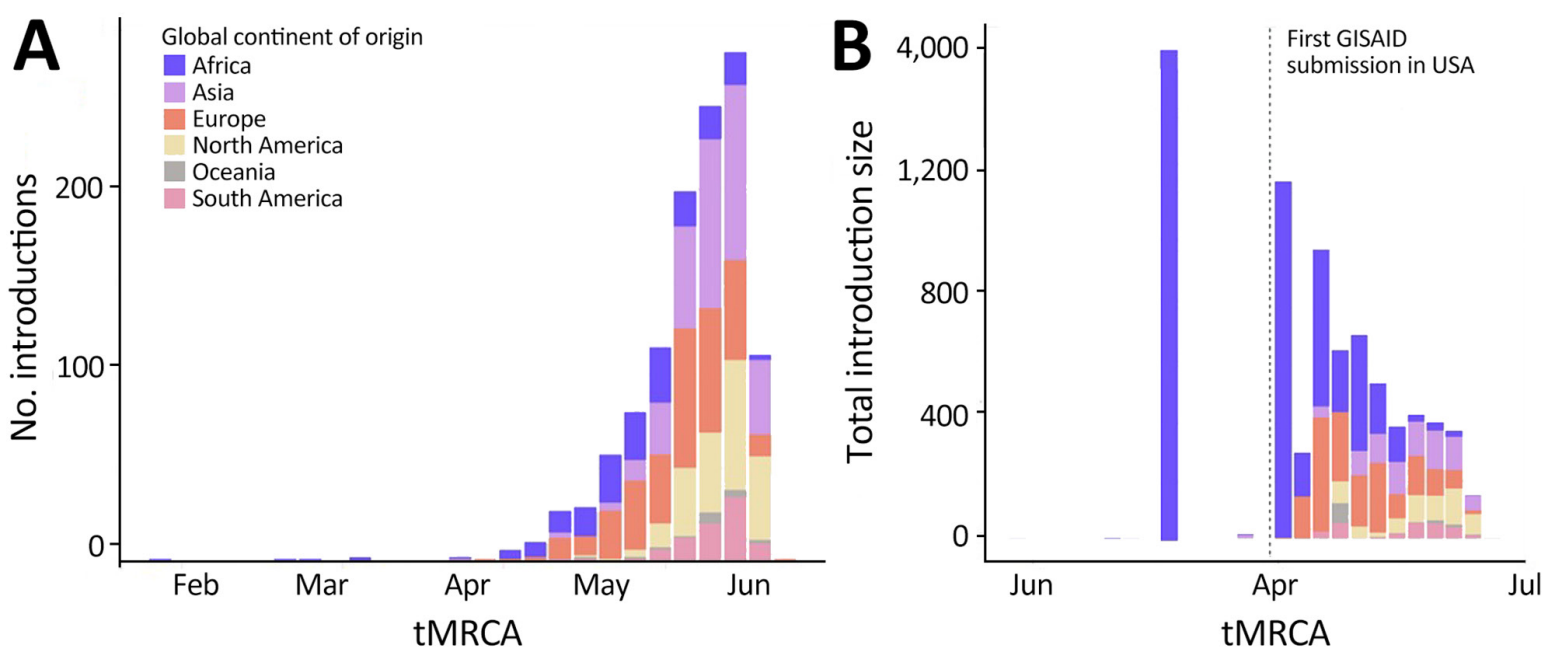
Spatiotemporal dynamics of international introductions of SARS-CoV-2 Omicron BA.5 lineage into the United States during February–June 2022. A) Numbers and timeline of BA.5 introduction events according to continent. B) Total introduction cluster size (number of sequences) of BA.5 international introduction events into the United States during the entire study period. Size was determined by the number of sequences per introduction. GISAID, https://www.gisaid.org; tMRCA, time to most recent common ancestor.

We examined the dynamics of BA.5 global introductions into the United States by using a discrete phylogeographic analysis at the continent level and between US regions (Figure 3) and reconstructed introductions across the resulting phylogeny (Figure 4). An average of 1,168 (95% CI 1,137–1,198) introductions occurred from other continents into the United States across the posterior distribution of the entire period (January 1, 2022, through the week of June 13, 2022). The inferred time of the first introduction into the United States was the second week of February 2022, nearly 3 weeks before the collection date of the earliest US sequence on February 26, 2022 (Figure 4). During the earlier part of this emergence period (until mid-May 2022), most (68%) introductions were from international importation (Figure 5), despite air travel in the United States being predominantly between US regions during the study period (domestic volume was ≈80% of all air travel volume) (Figure 6). After BA.5 became established in the United States in mid-May 2022 (Figure 2), 72% of the between-region introductions came from domestic sources (Figure 7). During the entire study period, most international introductions came from Asia (27.8%), Europe (26.3%), and Africa (14.7%) (Figure 8, panel A).

**Figure 9:**
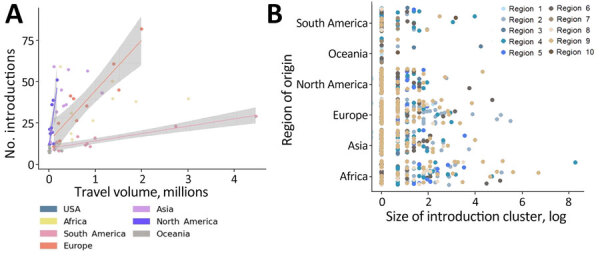
Associations between travel from different countries and number and cluster size of SARS-CoV-2 Omicron BA.5 introductions into the United States, February–June 2022. A) Linear regresssions indicating associations between the number of introductions into the United States from different continents and international travel volume according to that continent. (B) Cluster sizes of BA.5 introductions originating from different continents into the 10 Department of Health and Human Services regions of the United States. Regions designated by the US Department of Health and Human Services are shown in Figure 3.

We observed a chronological change in the relative dominance of continents as origins of BA.5 introductions into the United States (Figure 8). Introductions from Africa, despite only representing 14.7% of total BA.5 international introductions, comprised 41.9% of all international introductions before mid-May 2022. A high rate of introductions from Africa into all 10 US regions occurred, despite low travel volumes (Figure 9; Appendix Figures 2, 3). Indeed, Africa had the highest ratio of BA.5 introductions per travel volume, at ≈0.3 introductions per 1,000 passengers (Figure 9, panel A), likely because the BA.5 sublineage originated in Africa. As BA.5 prevalence increased globally, introductions from Europe, Asia, and North America became more critical (Figures 4, 5, 8), matching high travel volumes from those areas. Therefore, early emergence was determined by the variant’s geographic origin, whereas later introductions were connected to travel volume.

We examined the effect of timing on the size of international introduction events. During the first BA.5 introduction into the United States in early February 2022 and its detection ≈3 weeks later, 5 total introductions occurred (Figures 4, 8). Although 4 of those were singletons, 1 introduction from Africa during late February contained 3,980 sequences, the largest during the entire study period (Figures 8, panel B; Figure 9, panel B). Cluster size was highest during early introductions and decreased over time (Figure 8, panel B). Introduction events from Africa, most occurring earlier during the study period, tended to have high outbreak clade sizes; 9 clusters had >100 sequences (Figure 9, panel B). Introductions from Europe had only 4 clusters with >100 sequences; no other global regions had clusters of that size (Figure 9, panel B).

We found 2 main phases of BA.5 emergence in the United States. Large introductions from Africa dominated the early emergence phase before May 2022. As prevalence increased globally, international introductions had greater ties to air travel volume; hence, more introductions came from Europe, Asia, and North America. Because of a decrease in the susceptible population and possible behavior changes after an uptick in Omicron BA.5 cases, introductions from Europe, Asia, and North America did not expand as much as the earlier events from Africa.

### Domestic Movement of Omicron BA.5 in the United States 

To evaluate BA.5 transmission within the United States, we performed a discrete phylogeographic analysis using 10 DHHS-defined regions (Figure 3; Appendix Table 1). We inferred 3,137 within-country introductions across a single posterior tree, ≈70% of total introductions across the entire study period. Early international introduction events were followed by substantial domestic transmission (Figures 4, 5), and all 10 DHHS regions received >50% of their introductions from domestic sources (Figure 6). Those domestic movements grew in proportion throughout the study period and overtook the number of international introductions (Figures 5), aligning with the high (80%) proportion of domestic air travel (Figure 6).

**Figure 10:**
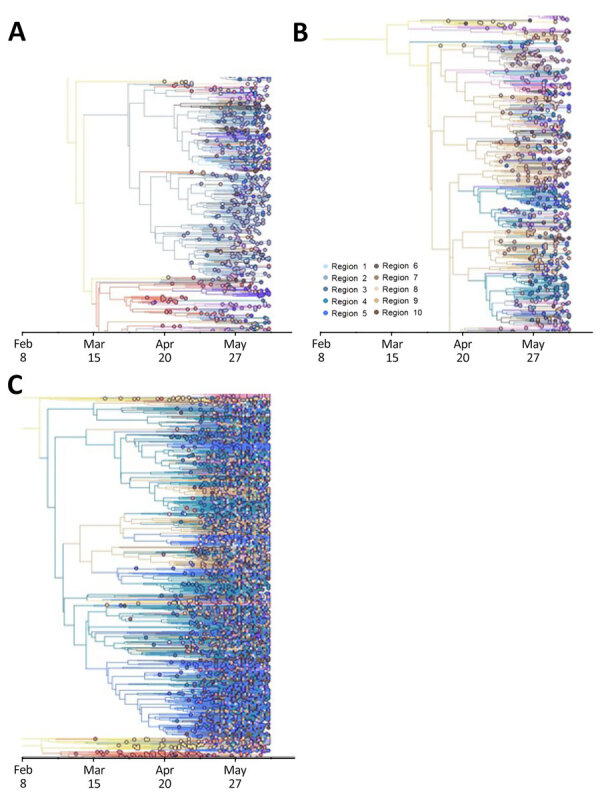
Time-scaled phylogenetic analysis of domestic SARS-CoV-2 Omicron BA.5 introductions between regions within the United States during February–June 2022. Phylogenies of the 3 largest and earliest US clades are indicated. Trees were rooted according to region 2 (A), region 9 (B), and region 4 (C). Regions designated by the US Department of Health and Human Services are shown in Figure 3.

**Figure 11:**
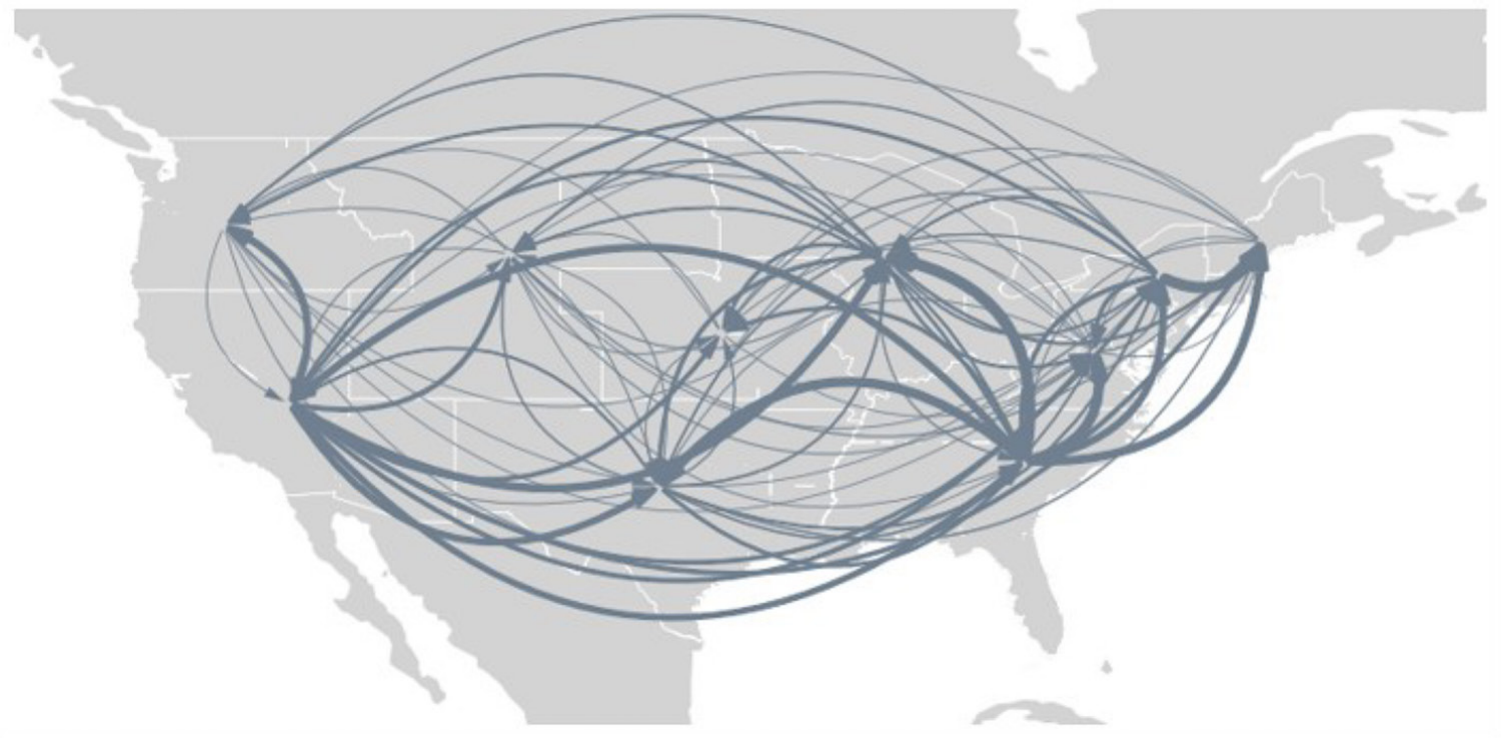
SARS-CoV-2 Omicron BA.5 movements between regions within the United States from study of BA.5 emergence during January–June 2022. Thickness of the lines indicates the prevalence of the movement across the maximum clade credibility tree; arrows indicate direction of introduction.

No noticeable geographic structure within the phylogeny was observed (i.e., sequences from different locations were intermixed, implying frequent interregion transmission during the emergence period) (Figure 4). Inspection of the 3 largest and earliest US clades, rooted in region 2 (several northeastern states, including New York), region 9 (southwestern states, including California), and region 4 (southeastern states, including Florida) (Figure 10), indicated that geographically close locations tended to have more interregion movement. Clades from region 2 and 4 were primarily transmitted to other East Coast regions, and clades from region 9 were transmitted to other West Coast and West/Central regions (Figures 10, 11). Nonetheless, interactions between regions 4 and 9, and to a lesser extent regions 2 and 5 (including Illinois), indicated coast-to-coast spread was a critical BA.5 emergence mechanism.

**Figure 12:**
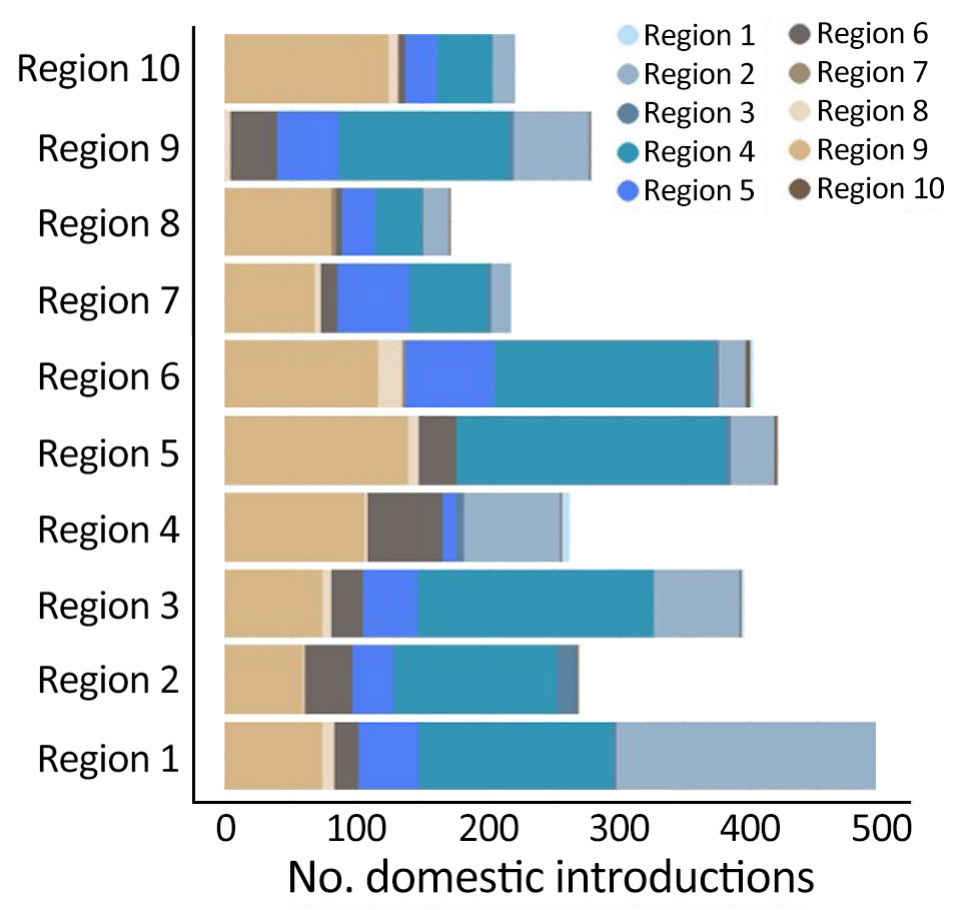
Number of domestic SARS-CoV-2 Omicron BA.5 introductions into each region of the United States in study of BA.5 emergence during January–June 2022. Regions designated by the US Department of Health and Human Services are indicated in Figure 3.

Several key hotspots for transmission existed. All DHHS regions had considerably higher introduction counts originating from regions 4 and 9 (Figure 12). The interaction rate between regions 4 and 9 and other regions represented 71.6% of total domestic BA.5 movements (Figure 12). Correspondingly, regions 4 and 9 also had the highest volumes of both international and domestic air travel (Figure 6). Region 1 (New England) had the highest (≈70%) number of incoming domestic introductions originating from regions 4 and 9 (Figure 12). Therefore, the strong transmission from regions 4 and 9 likely underpinned BA.5 emergence in the United States. We also theorize that region 1 was the top recipient of domestic introduction events because of the higher rate of interstate travel between regions 1–3, as well as incoming air travel from other regions (Figures 6, 11).

To explore possible underlying drivers of virus movement across the United States, we performed linear regressions between pairs of locations, using population sizes and whether those locations shared a border as predictors. We found that the population size of the origin location was a significant predictor for the number of virus movements between a pair of locations (p<0.0001). In comparison, the destination population combined with whether the 2 locations shared a land border was not a significant predictor for virus movement (p>0.1) (Appendix Table 2).

## Discussion

As SARS-CoV-2 continues to spread in the United States and globally, it will be essential to elucidate how new variants disseminate. We found that Omicron BA.5 was first introduced into the United States primarily from its geographic origin in Africa and then spread domestically from large populations and key hotspots, which are common between variants.

The earliest BA.5 introductions into the United States came from Africa despite low rates of air travel, indicating the importance of a variant’s geographic origin. Early introduction events were also much larger than later introductions, which is a common thread among the waves of SARS-CoV-2 across the globe, despite different demographic and intervention contexts (*13*). As prevalence rose globally in the later half of the study period, a higher proportion of introductions from Europe and Asia occurred, potentially corresponding to higher travel volume (*35*). Similar dynamics have occurred with Delta variant introductions into the United Kingdom (*11*). The combination of the earliest introductions being the most important and later introductions coming from many locations makes international travel restrictions challenging to implement, even aside from ethical concerns (*36*); the speed required to prevent the most critical early introductions from a particular origin, if it is even known, is unachievable in most settings.

Domestic transmission played a substantial role in BA.5 dissemination in the United States. Whereas rates of interregion transmission exceeded those of global importation across the entire study period, most domestic virus movement occurred during the later phase. We show widespread secondary transmission occurred across the United States after the initial international introduction, which corroborates previous findings indicating SARS-CoV-2 transmission is driven by domestic dynamics (*15*,*17*). The domestic BA.5 spread was significantly associated with population size of the origin location, which fits with previous descriptions of SARS-CoV-2 transmission starting from large urban centers into other areas (*37*,*38*). Along with geographic proximity being somewhat essential, that finding fits a classical gravity model of disease transmission (*39*).

Cross-country BA.5 spread between DHHS regions 2, 4, and 9 highlight the role of specific hotspots in promoting BA.5 emergence. Those 3 regions received the most introductions from Africa and had the 3 largest and earliest US clades, playing a critical role in receiving and disseminating early BA.5 introductions. That finding is similar to the dissemination of the SARS-CoV-2 Alpha variant (*17*); New York, New York, received the most introductions from the Alpha variant’s origin, followed by California and Florida. Therefore, we might expect those regions to be critical during future variant introductions. Furthermore, we found that region 1 (New England) was the highest recipient of domestic introductions, likely from high interaction rates with 2 of the key hotspots (regions 2 and 4). We suggest that regions 2, 4, and 9 were primary hotspots because of their major urban centers (e.g., New York, Atlanta, and Los Angeles). Those findings fit the description of early virus lineage movements between larger cities, followed by spatial expansion into nearby areas (*14*).

The first limitation of our study is that our subsampling method reflected the broader inequality in genomic surveillance worldwide (*22*,*23*). We attempted to minimize those biases through subsampling and categorization into broader continents and US DHHS regions. Rooting our tree in Africa, despite sequences from Europe overwhelming the global dataset, suggests that our attempts to mitigate this international bias were somewhat successful. Our categorization into larger regions (within and outside the United States) might have introduced residual confounding, preventing exploration of interstate introduction events. We also chose to use population size to subsample, rather than case-based metrics that might appear more relevant. However, obtaining unbiased incidence/hospitalization/death estimates during an outbreak is challenging, especially when comparing large geographic areas, such as the United States or entire continents. All data are imperfect sources of information in this context because large amounts of heterogeneity exist in how those data are recorded because of resource limitations, varying case definitions, and political concerns. We therefore used population size, which we concluded should be less biased. Second, geographic variation in sequencing efforts might have affected our cluster size results by artificially increasing the size of introductions from Africa compared with Europe (i.e., there might be missing sequences from Africa, which would split clusters into smaller introductions). Our downsampling scheme should have helped mitigate this limitation, and the pattern of early and large introductions fits with other settings (*13*). Third, we defined the variant emergence phase according to a frequency growth curve to filter for early BA.5 sequences, which we deemed essential to our research; that definition might not properly reflect the true emergence time for a novel variant, although this only changes the length of our study period. Finally, we did not test other factors that might have driven the international introduction of Omicron BA.5 into the United States, such as distance through air networks or income levels.

In conclusion, our findings support the role of phylogenetics in SARS-CoV-2 surveillance and contribute a phylogeographic framework for studying the emergence of other infectious pathogens in the United States. Countries have lifted pandemic restrictions and the general population has a mosaic of immunity; thus, the epidemiologic landscape presents opportunities for positive selection of novel SARS-CoV-2 variants. Determining the different dynamics of introduction in US regions will be critical for timely and cost-effective policymaking, particularly for health authorities. Our methods can be used to extend beyond SARS-CoV-2 analyses and can form a framework for phylogeographic analysis of large datasets to discern the spatiotemporal spread of other novel pathogens.

Appendix Additional information for large-scale genomic analysis of SARS-CoV-2 Omicron BA.5 emergence, United States.
