# Supplementary material for: Large-Scale Genomic Analysis of SARS-CoV-2 Omicron BA.5 Emergence, United States
Source: Emerg Infect Dis. 2025 May;31(Suppl 1):S45–56. doi: 10.3201/eid3113.240981 (PMC12078544; doi:10.3201/eid3113.240981)
Supplement: Appendix — Additional information for large-scale genomic analysis of SARS-CoV-2 Omicron BA.5 emergence, United States. [file 24-0981-Techapp-s1.pdf]

Article DOI: https://doi.org/10.3201/eid3113.240981

EID cannot ensure accessibility for supplementary materials supplied by authors. Readers who have difficulty accessing supplementary content should contact the authors for assistance.

Large-Scale Genomic Analysis of SARS-CoV-2 Omicron BA.5 Emergence, United States

Appendix

Appendix Table 1. Components of United States DHHS regions*

DHHS region	US states
1	Connecticut, Maine, Massachusetts, New Hampshire, Rhode Island, Vermont
2	New York, New Jersey, Puerto Rico, Virgin Islands
3	Delaware, Maryland, Pennsylvania, Virginia, West Virginia, District of Columbia
4	Alabama, Florida, Georgia, Kentucky, Mississippi, North Carolina, South Carolina, Tennessee
5	Illinois, Indiana, Ohio, Michigan, Minnesota, Wisconsin
6	Arkansas, Louisiana, New Mexico, Oklahoma, Texas
7	Iowa, Kansas, Missouri, Nebraska
8	Colorado, Montana, North Dakota, South Dakota, Utah, Wyoming
9	Arizona, California, Hawaii, Nevada, Guam, Trust Territory of the Pacific Islands, American Samoa
10	Alaska, Idaho, Oregon, Washington

*Regions were designated by DHHS (https://www.hhs.gov/about/agencies/ogc/offices/regional-offices/index.html). DHHS, Department of Health and Human Services.

Appendix Table 2. Results of generalized linear model comparing the number of introductions between pairs of locations with whether they share a border or the human population size of the origin and destination

Coefficient	Coefficient estimate	Standard error	p value
Intercept	−1.366	0.3440	0.000148
Neighbor, yes/no	0.3573	0.2446	0.1477
Population origin	5.964×10^{-8}	6.391×10^{-9}	1.04×10^{-14}
Population destination	1.005×10^{-8}	6.412×10^{-9}	0.1207

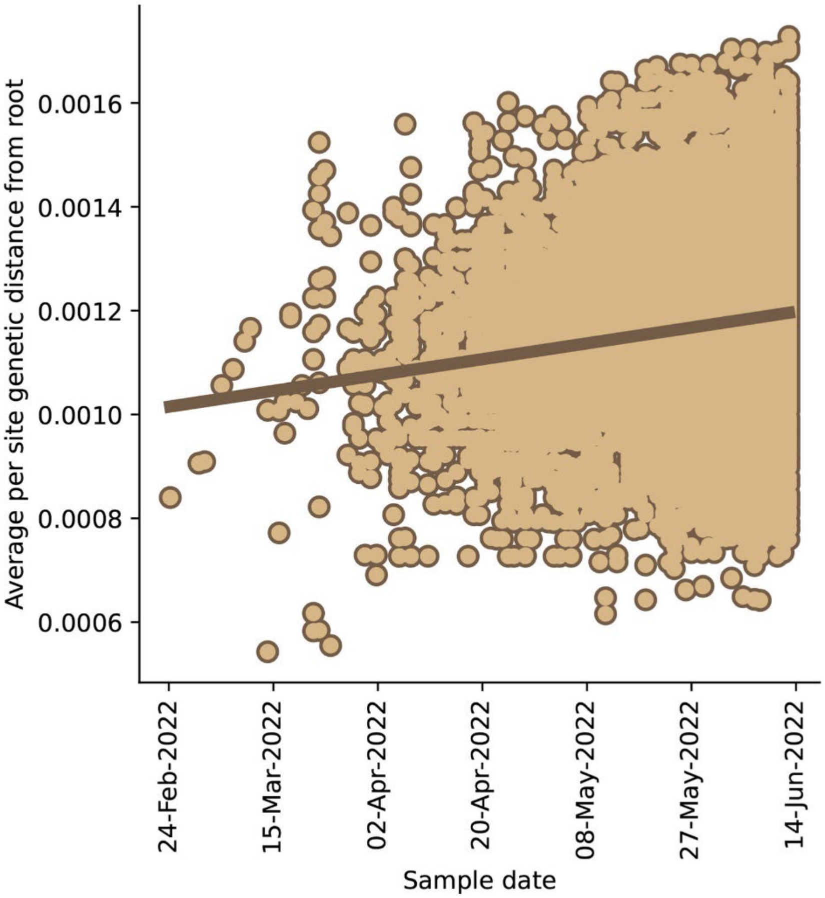

Appendix Figure 1. Root-to-tip regression plot indicating insufficient temporal signal in dataset of SARS-CoV-2 BA.5 sequences to estimate the rate of evolution. Rate was fixed in subsequent analyses.

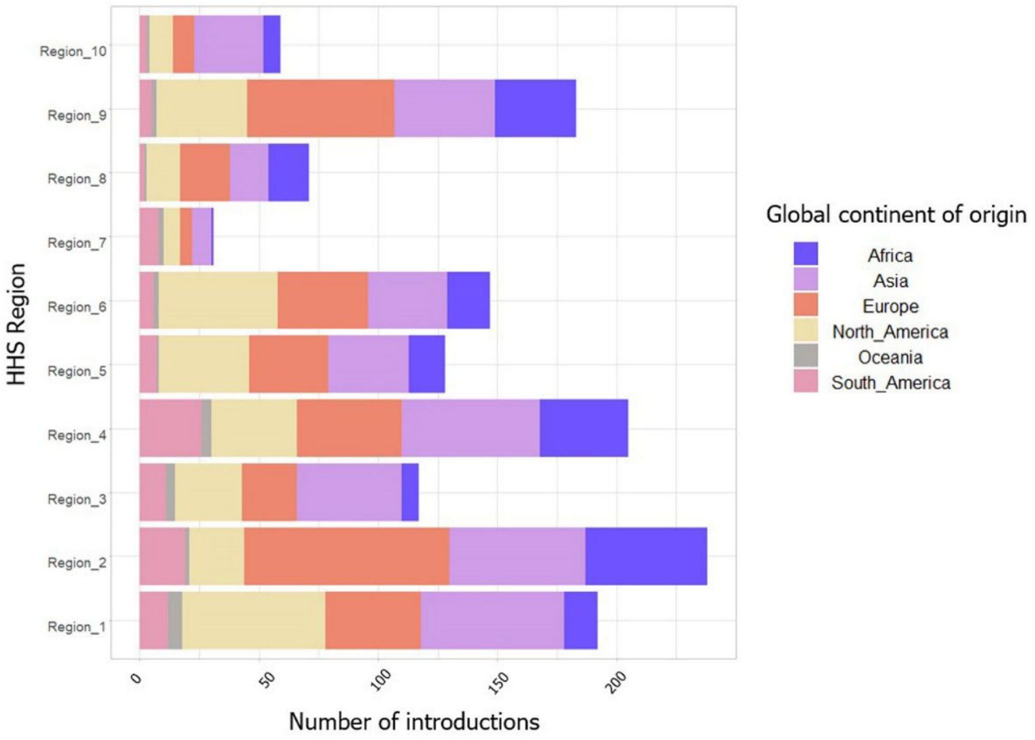

Appendix Figure 2. Number of international introductions of SARS-CoV-2 Omicron BA.5 into 10 Department of Health and Human Services regions of the United States during January–June 2022 according to continent. HHS, Health and Human Services.

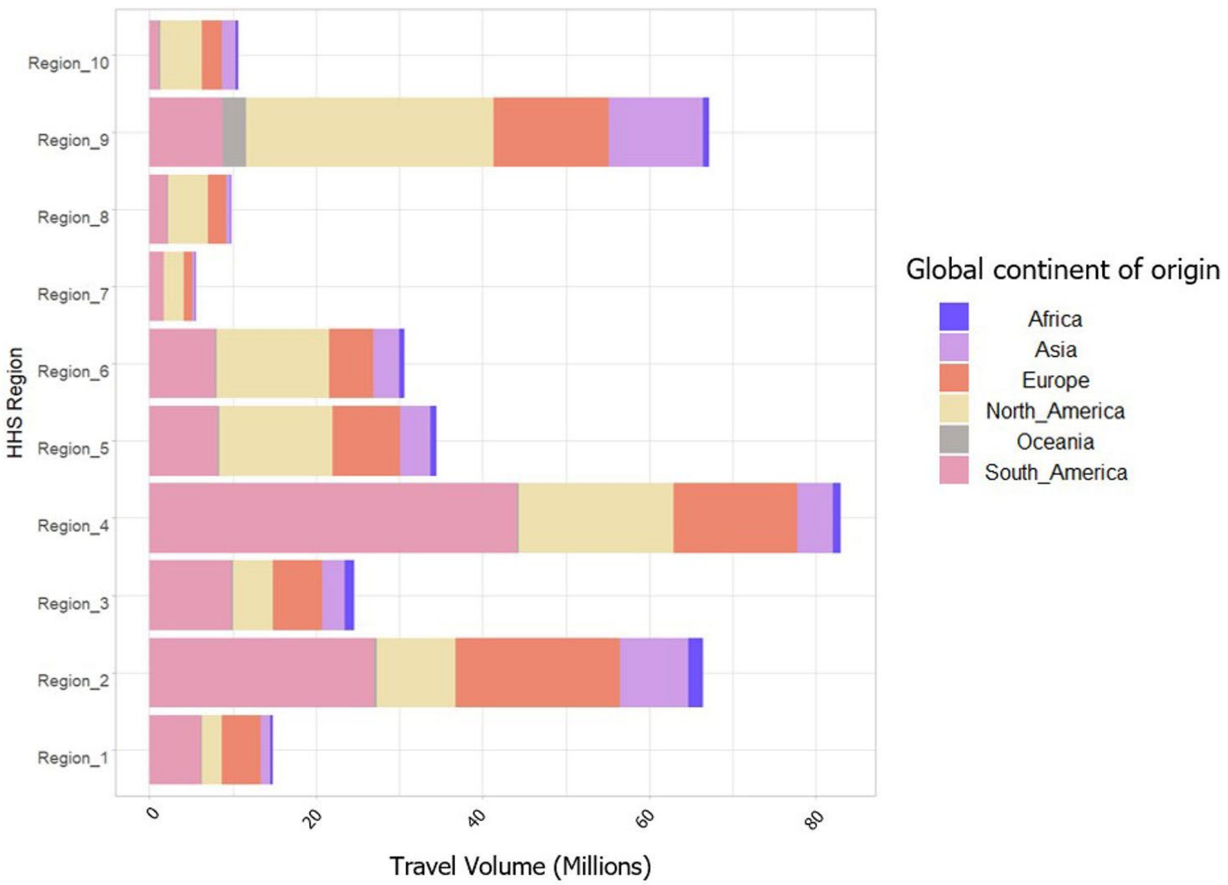

Appendix Figure 3. International travel volume into 10 Department of Health and Human Services regions of the United States during January–June 2022 according to continent. HHS, Health and Human Services.
